# Temporal Trends in Cervical Spine Curvature of South Korean Adults Assessed by Deep Learning System Segmentation, 2006-2018

**DOI:** 10.1001/jamanetworkopen.2020.20961

**Published:** 2020-10-15

**Authors:** YiRang Shin, Kyunghwa Han, Young Han Lee

**Affiliations:** 1 Department of Radiology, Research Institute of Radiological Science, Center for Clinical Imaging Data Science, Yonsei University College of Medicine, Seoul, South Korea

## Abstract

**Importance:**

The loss of the physiologic cervical lordotic curve is a common degenerative disorder known to be associated with abnormal spinal alignment. However, the changing trends among sex and age groups has not yet been well established.

**Objective:**

To analyze the temporal trends in cervical curvature across sex and age groups using an automated deep learning system (DLS).

**Design, Setting, and Participants:**

A retrospective cross-sectional study was conducted using lateral cervical radiographs of 13 691 individuals from January 1, 2006, to December 31, 2018. The degree of anterior vertical curvature was approximated by a DLS approach and convexity measurement method. This population-based study used the Yonsei University College of Medicine Severance Hospital, Seoul, South Korea, cohort database to identify 13 691 consecutive adults (≥18 years of age) who underwent standing lateral radiography in inpatient and outpatient settings.

**Main Outcomes and Measures:**

The prevalence of kyphotic and straight cervical curve as well as the trends of degree of cervical curvature in 2006 to 2018 among sex and age groups were determined. The DLS performance was validated with quantitative metrics and compared with interobserver and intraobserver variations.

**Results:**

Automatic cervical spine segmentation was identified from lateral radiographs of 13 691 individuals (mean [SD] age, 49.9 [15.3] years; 8051 women [58.8%]). From 2006 to 2018, the decrease in the lordotic curve was significant across both sexes and age groups younger than 70 years, with the decrease more pronounced in women and successively younger generations (female, −0.05; 95% CI, −0.06 to −0.04; 18-29 years of age, −0.06; 95% CI, −0.08 to −0.04; 30-39 years of age, −0.06; 95% CI, −0.08 to −0.04; and 40-49 years of age, −0.05; 95% CI, −0.06 to −0.03; all *P* < .001). The prevalence of straight and kyphotic curvature had a significant increasing trend for both sexes and young generations, in which individuals 18 to 29 years of age generally had the highest prevalence rates during the study cycle (in 2018, kyphosis, 16.7%; 95% CI, 10.8%-22.5%; straight, 45.5%; 95% CI, 37.7%-53.3%). Similar trends were observed with longitudinal analysis of repeated measures of individuals, with more pronounced decreases in lordotic curvature observed among women and young adults.

**Conclusions and Relevance:**

This study suggests a significant, increasing loss of normal cervical lordotic curvature for both sexes and young adults that is greater in progressively younger cohorts and women. Further research is necessary to evaluate associations between neck pain and loss of cervical curvature and address the need for active promotion and practical interventions aimed at neck posture correction.

## Key Points

**Question:**

How has the curvature of the cervical spine changed among sex and age groups in South Korea?

**Findings:**

In this cross-sectional study, the cervical curvature of 13 691 individuals was measured through a deep learning system. From 2006 to 2018, the prevalence of kyphotic and straight curvature increased significantly for both sexes and young adults, with the corresponding decrease of lordotic cervical curvature being more pronounced in successively younger generations and women.

**Meaning:**

A novel deep learning system was used to investigate large-scale trends of the cervical curvature. Lordotic cervical curvature significantly decreased across both sexes and all age groups younger than 70 years between 2006 and 2018.

## Teaser

This cross-sectional study uses a deep learning system to analyze the temporal trends in cervical spine curvature across sex and age groups in a population of South Korean adults.

## Introduction

The internet and mobile devices are widely used in modern society, affecting daily life in the workplace and at home. In 2016, internet use on mobile devices outpaced that on desktop computers^1^; thus, people more commonly access the internet while active than while stationary. The rapid increase in the use of mobile devices and computers has resulted in increased repetitive or prolonged postures, neck flexion,^2^ and mechanical loading in the cervical spine.^3^ Prolonged use of mobile devices is known to aggravate the degree and severity of neck and shoulder pain (NSP).^4,5^ In 2010, NSP became the fourth leading cause of significant musculoskeletal disability worldwide,^6^ with an annual prevalence rate exceeding 30%.^7^

Corresponding to the increase in static postures in the modern sedentary lifestyle, the skeletal shape of humans has changed during the past decade.^8,9^ The prolonged use of mobile devices leads to deviations from the neutral head postures and induces deeper neck flexion,^10,11^ increasing concerns that use of mobile devices may have facilitated spinal misalignment and degeneration. Loss of cervical lordosis is a common disorder of the cervical spine that results in loss or reversal of the normal curvature. Previous cross-sectional studies have examined the specific associations between the degree of cervical lordosis and sex^12,13^ and age.^14,15^ However, studies have not reported trends over time because manual measurement of the cervical curvature is time-consuming and labor intensive.

To address these trends, we used artificial intelligence (AI) to automatically analyze large-scale radiographs to assess the prevalence of loss of lordotic cervical curvature. Since the introduction of the picture archiving and communication system during the past 2 decades, vast quantities of radiologic images stored in servers have allowed the field of radiology to benefit from big data analysis techniques.^16^ Artificial intelligence–based deep learning has demonstrated promising diagnostic performance for various applications, including lesion detection in radiologic images,^17,18^ classification in breast cancer,^19^ and diabetic retinopathy.^20^

This analysis for cervical curvature in South Korea represents the largest epidemiologic study, to our knowledge, to document cervical vertebral curvature associated with age and sex during 13 years. This study analyzes the temporal trends in cervical curvature across sex and age groups using an automated deep learning system (DLS).

## Methods

### Study Population

**Figure 1.**
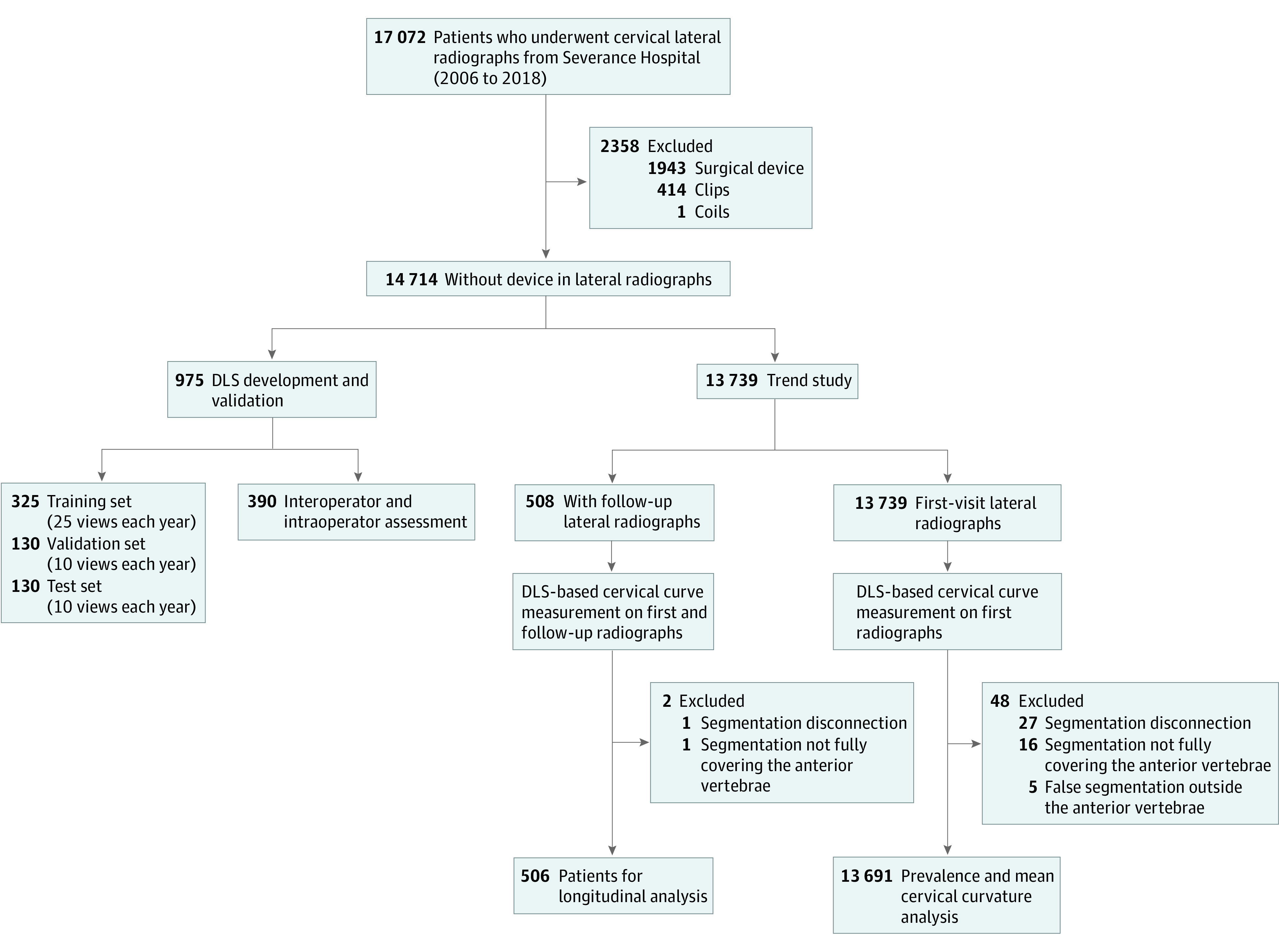
Flow of Inclusion Process of Lateral Radiographs From the Severance Hospital From 2006 to 2018 DLS indicates deep learning system.

This cross-sectional study included lateral view radiographs of the cervical spine of adult patients (≥18 years of age) in inpatient and outpatient settings between January 1, 2006, and December 31, 2018. The data were retrieved from the picture archiving and communication system of Severance Hospital, Yonsei University College of Medicine, in Seoul, South Korea. The detailed inclusion and exclusion criteria are given in eMethods 1 in the Supplement. Overall, 15 301 radiographs from 14 714 unique patients were used for this study; 975 radiographs (from 975 unique patients) were selected to develop and validate the DLS model, 13 739 radiographs of first-visit patients were used for trend analysis, and 1095 radiographs were collected from 508 patients with follow-up radiographs for longitudinal analysis. For the inclusion of a cervical curvature epidemiologic study, a qualitative review was performed on the DLS-segmented anterior vertebral line to exclude the cases with segmentation error. The exclusion criteria are summarized in eMethods 2 in the Supplement. The final analytic population demographic characteristics are listed in Table 1. The flowchart of the entire inclusion process is presented in Figure 1. The study was approved by the institutional review board of Severance Hospital, Yonsei University College of Medicine. The data were anonymized before analysis, and because of the retrospective nature of this study, the need for individual consent was waived. This study followed the Strengthening the Reporting of Observational Studies in Epidemiology (STROBE) reporting guideline.^21^

**Table 1.** Patient Demographic Characteristics

Demographic	Year												
	2006	2007	2008	2009	2010	2011	2012	2013	2014	2015	2016	2017	2018
**First-visit patient demographic characteristics for mean cervical curvature and prevalence trend analysis**													
No. of images	970	1239	1038	899	621	473	973	990	980	1408	1243	1655	1202
No. (%) of women/No. of individuals	550/970 (56.7)	706/1239 (57.0)	576/1038 (55.5)	520/899 (57.8)	351/621 (56.5)	299/473 (63.2)	571/973 (58.7)	575/990 (58.1)	556/980 (56.7)	869/1408 (61.7)	780/1243 (62.8)	982/1655 (59.3)	716/1202 (59.6)
Age, mean (SD), y													
Women	50.3 (14.0)	50.5 (13.7)	51.1 (14.6)	49.8 (15.3)	50.4 (13.9)	52.6 (13.4)	51.0 (14.9)	50.3 (14.7)	49.9 (15.9)	51.3 (14.5)	51.3 (14.5)	52.0 (14.7)	51.5 (15.3)
Men	47.8 (15.1)	47.2 (15.9)	47.9 (15.6)	47.6 (16.4)	49.8 (15.3)	50.7 (16.1)	46.6 (15.4)	46.1 (15.6)	47.9 (16.0)	48.9 (16.3)	51.4 (16.5)	49.6 (16.6)	49.4 (17.1)
**Demographic characteristics for patients with follow-up for longitudinal analysis**													
Mean (SD) No. of follow-up images	2.1 (0.4)	2.3 (0.8)	2.1 (0.5)	2.1 (0.7)	2.1 (0.5)	2.2 (0.5)	2.1 (0.5)	2.2 (0.5)	2.2 (0.4)	2.1 (0.4)	2.1 (0.3)	2.0 (0.0)	NA
No. (%) of women/No. of individuals at first visit	45/83 (54.2)	39/69 (56.5)	36/63 (57.1)	20/37 (54.1)	21/36 (58.3)	26/33 (78.8)	18/24 (75.0)	10/22 (45.5)	18/39 (46.2)	20/34 (58.8)	18/35 (51.4)	19/31 (61.3)	NA
Age, mean (SD), y													
First visit	52.7 (11.6)	53.5 (12.3)	52.0 (13.5)	53.1 (13.5)	53.3 (12.4)	52.4 (13.0)	55.9 (11.2)	45.8 (12.8)	54.1 (14.9)	55.4 (13.9)	49.8 (14.9)	55.6 (13.8)	NA
Final visit	56.9 (12.2)	58.6 (12.4)	55.7 (13.3)	56.5 (13.8)	56.3 (13.0)	56.2 (13.3)	59.4 (10.8)	48.6 (12.7)	56.5 (14.7)	57.2 (13.8)	51.3 (14.9)	56.6 (13.8)	NA

Abbreviation: NA, not applicable.

### Development and Validation of the DLS

The DLS model for anterior vertical line segmentation on lateral radiographs was trained on the basis of U-Net architecture, a type of convolutional neural network that demonstrated a powerful performance in the segmentation of medical images.^22^ A total of 325 images were selected randomly (25 images per year) for training. A subset of 130 images (10 images per year) were selected as a validation set and test set after being reviewed by a board-certified radiologist (Y.H.L.). The patient demographic characteristics of the training, validation, and test sets are listed in eTable 1 in the Supplement. The proposed DLS network for extracting the anterior vertical lines with technical details is illustrated in eFigure 1 and eMethods 3 in the Supplement.

For the quantitative validation of the proposed DLS, we performed a reliability test using pixel-wise accuracy and mean intersection over the union metrics (a mean of the correct pixel classification divided by the total number of pixels of that class metrics).^23^ In addition, the level of error was assessed using interoperator and intraoperator investigations on 390 reviewed radiographs (30 radiographs per year). To evaluate the consistency between the manually measured curvature and the DLS-predicted anterior vertebral line, we used the root-mean-squared error and the intraclass correlation coefficient.

### Measurement of Cervical Spine Curvature on DLS Segmentation

The curvature (from convex to concave) of the segmented cervical vertebral line of the DLS model was evaluated using polynomial regression, following the methods described by Tecco and Festa^13^ and Ferrario et al.^24^ The x-axis was superimposed on the DLS segmented vertebral line (eFigure 2 in the Supplement). The midpoint of this line was set as origin. The y-axis was drawn perpendicular to the x-axis, intersecting the origin. The digitized coordinates of the anterior margin of the vertebral bodies are interpolated by a second-order polynomial function (*y* = *a* + *bx* + *cx*^2^) to determine the convexity (or concavity) of the cervical curvature. The convexity (or concavity) of the cervical column is expressed as *c* × 10^3^; the larger the absolute value, the more convex (or concave) the curve. Conventionally, the curve is lordotic when the sign of coefficient *c* is positive, kyphotic when it is negative, and straight when the value is zero. Python, version 3.5.6 (Python Software Foundation) was used for digitizing the points, calculating the cartesian coordinates, and interpolating the curve.

The kyphotic, straight, and lordotic groups of the cervical curves were determined by converting the obtained coefficient to existing measurement methods validated by previous studies.^25,26,27^ The four-line Cobb method and the Harrison posterior tangent method are widely acknowledged reliable methods.^25^ The Harrison posterior tangent method was used because of a lower SEM compared with that of the Cobb method. An expert radiologist (Y.H.L.) measured the angle between the posterior wall of the vertebral bodies of C2 and C7. On the basis of the global angle cutoffs determined in previous studies (lordosis: <−4°, straight: −4° to +4°, and kyphosis: >+4°)^26,28^ and converting the angles to the proposed cervical curvature coefficient, the patients were classified into lordotic, straight, and kyphotic groups (eFigure 3 in the Supplement). The corresponding cervical coefficient cutoffs to represent the lordotic, straight, and kyphotic groups are as follows: straight: −0.8 to 0.8, lordotic: more than 0.8, and kyphotic: less than −0.8.

### Statistical Analysis

The means and SDs of the cervical curve coefficient were calculated by sex and age for 2006 to 2018. Linear regression analysis was performed to examine the trend of mean cervical curvature to estimate the regression coefficients with the associated 95% CIs. The age-specific rates were calculated in 10-year age groups (ie, 18-29, 30-39, 40-49, 50-59, 60-69, and ≥70 years).

The prevalence estimates of kyphotic and straight curvature were each calculated for both sex and all age groups for 2006 to 2018. The significance of the trend in each kyphotic and straight cervical curvature group was tested using multinomial logistic regression across the study cycle to estimate the regression coefficients (β) and 95% CIs for the changes. The independent variables included in the model were year, sex, and age.

In addition, cases repeatedly measured in the same individuals were incorporated to analyze the change of cervical curvature over time (per year) by sex and age groups. We applied a linear mixed-effects model to analyze the trend of individual cervical coefficients. The year, sex, and age were considered fixed effects, and the random slope on year was added in the model. No data were missing. The *P* value for each term tests the null hypothesis that the coefficient is equal to 0, and 2-sided *P* < .05 was considered statistically significant. All statistical analyses were performed using R, version 3.6.2 (R Foundation for Statistical Computing).

## Results

### Study Population

Automatic cervical spine segmentation was identified from lateral radiographs of 13 691 individuals (mean [SD] age, 49.9 [15.3] years; 8051 women [58.8%]). The final analytic sample was 13 691 radiographs from 13 691 first-visit patients and 1091 radiographs from 506 patients with repeated measures.

### DLS Accuracy

The segmentation performance of the DLS was evaluated on the test set. The performance of DLS segmentation for the anterior vertebral line had a pixel-wise accuracy of 96.67% and a mean intersection over the union of 85.72%. In the qualitative review, 14 276 DLS segmentations were confirmed to have successfully drawn the anterior vertebral line along the anterior margin of the C1/2 to C6/7 vertebral bodies (n = 14 276 of 14 326 [99.65%]). Mislabeled segmentations (n = 50 [0.35%]) were excluded from the trend analysis.

In addition, 390 cervical curvature estimates (30 images per year) annotated by an expert radiologist (Y.H.L.) and predicted by DLS were compared (eTable 2 in the Supplement). The intraclass correlation coefficient of measurements for each year yielded high-reliability coefficients (ranging from 0.974 to 0.993), which indicates significant consistency between curvature estimates of expert radiologist (Y.H.L.) annotations and DLS outputs. The root-mean-squared error for the cervical curve between the 2 observers was less than 0.30 throughout the study cycle.

### Trend of Loss of Normal Cervical Curve

Overall, the mean (SD) of the cervical curvature decreased from 1.76 (1.37) to 1.24 (1.37), highlighting the trend of the loss of lordotic cervical curve from 2006 to 2018 (eTable 3 in the Supplement). From 2006 to 2018, the estimated prevalence of straight curvature increased from 24.8% (95% CI, 20.6%-28.9%) to 32.0% (95% CI, 27.9%-36.2%) for men and from 16.5% (95% CI, 13.4%-19.7%) to 26.9% (95% CI, 23.6%-30.1%) for women (all *P* < .001). For kyphotic curvature, the prevalence increased from 2.9% (95% CI, 1.3%-4.5%; *P* = .004) to 4.7% (95% CI, 2.8%-6.6%; *P* = .004) for men and from 2.7% (95% CI, 1.4%-4.1%; *P* < .001) to 7.8% (95% CI, 5.9%-9.8%; *P* < .001) for women (Table 2). Men were more prone to have a higher prevalence of abnormal (kyphotic and straight) cervical curvature compared with women; however, the difference between men and women decreased from 8.5% (2006) to 2.0% (2018).

**Table 2.** Annual Trends in the Prevalence of Kyphotic and Straight Cervical Curvature by Age and Sex, 2006 to 2018

Demographic	Prevalence, % (95% CI)													β (95% CI)	*P* value for trend
	2006	2007	2008	2009	2010	2011	2012	2013	2014	2015	2016	2017	2018		
**Prevalence of straight cervical curvature**															
Sex															
Male	24.8 (20.6 to 28.9)	25.3 (21.6 to 29.0)	28.0 (23.9 to 32.0)	26.2 (21.8 to 30.6)	30.3 (24.9 to 35.7)	30.2 (23.5 to 36.9)	25.0 (20.8 to 29.2)	27.8 (23.5 to 32.1)	31.5 (27.0 to 35.9)	27.7 (23.9 to 31.4)	33.0 (28.8 to 37.2)	35.0 (31.4 to 38.6)	32.0 (27.9 to 36.2)	0.04 (0.02 to 0.05)	<.001
Female	16.5 (13.4 to 19.7)	18.7 (15.8 to 21.6)	20.8 (17.5 to 24.1)	22.5 (18.9 to 26.1)	20.2 (16.0 to 24.3)	20.7 (16.1 to 25.3)	25.8 (22.2 to 29.3)	24.8 (21.3 to 28.4)	26.5 (22.9 to 30.2)	24.6 (21.8 to 27.5)	28.9 (25.7 to 32.1)	28.8 (26.0 to 31.7)	26.9 (23.6 to 30.1)	0.06 (0.04 to 0.07)	<.001
Age range, y															
18-29	34.8 (26.0 to 43.6)	37.9 (30.4 to 45.4)	37.2 (28.9 to 45.6)	42.7 (34.0 to 51.4)	43.3 (31.4 to 55.1)	35.9 (20.8 to 51.0)	41.5 (33.2 to 49.8)	42.3 (33.5 to 51.0)	50.7 (42.3 to 59.2)	41.2 (33.3 to 49.1)	48.1 (39.4 to 56.7)	45.4 (38.2 to 52.6)	45.5 (37.7 to 53.3)	0.06 (0.03 to 0.08)	<.001
30-39	26.3 (18.8 to 33.8)	22.6 (16.4 to 28.8)	36.4 (28.5 to 44.2)	35.1 (27.0 to 43.2)	32.1 (21.9 to 42.3)	39.0 (26.5 to 51.4)	33.1 (25.4 to 40.8)	37.7 (30.2 to 45.1)	37.4 (29.8 to 45.0)	37.8 (31.3 to 44.2)	40.6 (33.2 to 48.0)	42.1 (35.7 to 48.5)	40.6 (33.0 to 48.2)	0.07 (0.04 to 0.09)	<.001
40-49	21.5 (16.1 to 26.9)	26.2 (20.8 to 31.5)	23.4 (17.7 to 29.0)	26.7 (19.9 to 33.5)	27.7 (20.0 to 35.4)	34.5 (24.4 to 44.7)	29.9 (23.1 to 36.7)	27.1 (21.0 to 33.2)	32.8 (25.9 to 39.7)	27.3 (22.0 to 32.5)	38.6 (32.2 to 45.1)	35.0 (29.6 to 40.4)	36.0 (29.3 to 42.7)	0.06 (0.04 to 0.08)	<.001
50-59	17.3 (12.6 to 22.0)	17.0 (12.8 to 21.2)	22.1 (17.1 to 27.2)	18.7 (14.1 to 23.3)	17.5 (11.9 to 23.1)	19.9 (13.6 to 26.1)	21.1 (16.2 to 26.0)	19.4 (14.6 to 24.2)	21.4 (16.4 to 26.3)	20.7 (16.5 to 24.9)	28.1 (23.3 to 32.8)	29.1 (24.7 to 33.5)	25.7 (20.7 to 30.7)	0.05 (0.03 to 0.07)	<.001
60-69	12.4 (7.5 to 17.2)	13.4 (9.2 to 17.7)	16.5 (11.6 to 21.4)	9.6 (4.8 to 14.4)	21.4 (14.4 to 28.4)	13.0 (6.2 to 19.9)	14.7 (9.4 to 20.0)	16.7 (10.8 to 22.5)	16.0 (10.3 to 21.8)	19.8 (15.1 to 24.5)	22.6 (17.4 to 27.8)	23.7 (19.1 to 28.2)	18.4 (13.5 to 23.2)	0.05 (0.03 to 0.08)	<.001
≥70	10.7 (3.7 to 17.7)	14.8 (7.4 to 22.2)	9.8 (3.3 to 16.2)	14.7 (6.3 to 23.1)	11.1 (1.9 to 20.3)	15.7 (5.7 to 25.7)	13.6 (6.5 to 20.8)	15.9 (7.9 to 23.8)	16.7 (9.2 to 24.1)	13.3 (7.7 to 18.9)	9.7 (4.9 to 14.6)	17.7 (12.3 to 23.1)	13.5 (8.0 to 19.0)	0.02 (−0.02 to 0.06)	.43
**Prevalence of kyphotic cervical curvature**															
Sex															
Male	2.9 (1.3 to 4.5)	4.5 (2.7 to 6.2)	5.3 (3.3 to 7.3)	2.8 (1.2 to 4.5)	3.3 (1.2 to 5.4)	4.9 (1.8 to 8.1)	4.5 (2.4 to 6.5)	3.6 (1.8 to 5.4)	5.6 (3.4 to 7.8)	4.2 (2.5 to 5.8)	4.8 (2.9 to 6.7)	6.8 (4.9 to 8.7)	4.7 (2.8 to 6.6)	0.05 (0.02 to 0.08)	.004
Female	2.7 (1.4 to 4.1)	2.7 (1.5 to 3.9)	3.6 (2.1 to 5.2)	1.9 (0.7 to 3.1)	5.3 (3.0 to 7.7)	4.7 (2.3 to 7.1)	4.2 (2.5 to 5.8)	4.7 (3.0 to 6.5)	6.3 (4.3 to 8.3)	3.8 (2.5 to 5.1)	4.8 (3.3 to 6.3)	6.0 (4.5 to 7.5)	7.8 (5.9 to 9.8)	0.09 (0.06 to 0.12)	<.001
Age range, y															
18-29	4.5 (0.6 to 8.3)	6.8 (2.9 to 10.7)	10.1 (4.9 to 15.3)	7.3 (2.7 to 11.8)	13.4 (5.3 to 21.6)	12.8 (2.3 to 23.3)	9.6 (4.7 to 14.6)	13.0 (7.1 to 19.0)	14.2 (8.3 to 20.1)	12.2 (6.9 to 17.4)	12.4 (6.7 to 18.1)	14.6 (9.5 to 19.7)	16.7 (10.8 to 22.5)	0.11 (0.06 to 0.15)	<.001
30-39	3.0 (0.1 to 5.9)	6.2 (2.7 to 9.8)	8.4 (3.8 to 12.9)	2.2 (0.0 to 4.7)	6.2 (0.9 to 11.4)	10.2 (2.5 to 17.9)	7.6 (3.3 to 11.9)	6.8 (2.9 to 10.7)	12.3 (7.1 to 17.4)	7.4 (3.9 to 10.9)	8.2 (4.1 to 12.4)	12.3 (8.0 to 16.5)	11.9 (6.9 to 16.9)	0.11 (0.06 to 0.16)	<.001
40-49	3.1 (0.9 to 5.4)	1.9 (0.3 to 3.6)	1.9 (0.1 to 3.7)	5.0 (1.6 to 8.3)	3.8 (0.5 to 7.2)	2.4 (0.0 to 5.6)	5.2 (1.9 to 8.5)	5.4 (2.3 to 8.5)	6.2 (2.7 to 9.8)	3.3 (1.2 to 5.4)	4.5 (1.8 to 7.3)	8.3 (5.2 to 11.3)	6.6 (3.1 to 10.1)	0.11 (0.06 to 0.16)	<.001
50-59	2.4 (0.5 to 4.3)	1.6 (0.2 to 3.0)	3.1 (1.0 to 5.1)	0.0 (0.0 to 0.0)	2.3 (0.1 to 4.4)	1.9 (0.0 to 4.1)	2.2 (0.5 to 4.0)	1.1 (0.0 to 2.4)	1.5 (0.0 to 3.0)	2.0 (0.5 to 3.4)	3.2 (1.3 to 5.1)	2.9 (1.3 to 4.5)	3.0 (1.1 to 5.0)	0.06 (0.00 to 0.12)	.06
60-69	2.2 (0.1 to 4.4)	3.3 (1.0 to 5.5)	2.3 (0.3 to 4.3)	0.7 (0.0 to 2.0)	2.3 (0.0 to 4.9)	6.5 (1.5 to 11.6)	0.6 (0.0 to 1.7)	0.6 (0.0 to 1.9)	3.2 (0.4 to 6.0)	1.1 (0.0 to 2.3)	2.0 (0.3 to 3.8)	2.4 (0.8 to 4.0)	3.7 (1.3 to 6.0)	0.01 (−0.05 to 0.08)	.73
≥70	1.3 (0.0 to 3.9)	3.4 (0.0 to 7.2)	4.9 (0.2 to 9.5)	0.0 (0.0 to 0.0)	4.4 (0.0 to 10.5)	2.0 (0.0 to 5.8)	2.3 (0.0 to 5.4)	0.0 (0.0 to 0.0)	1.0 (0.0 to 3.1)	2.1 (0.0 to 4.4)	2.8 (0.1 to 5.5)	2.6 (0.4 to 4.9)	2.0 (0.0 to 4.3)	−0.01 (−0.11 to 0.08)	.78

**Figure 2.**
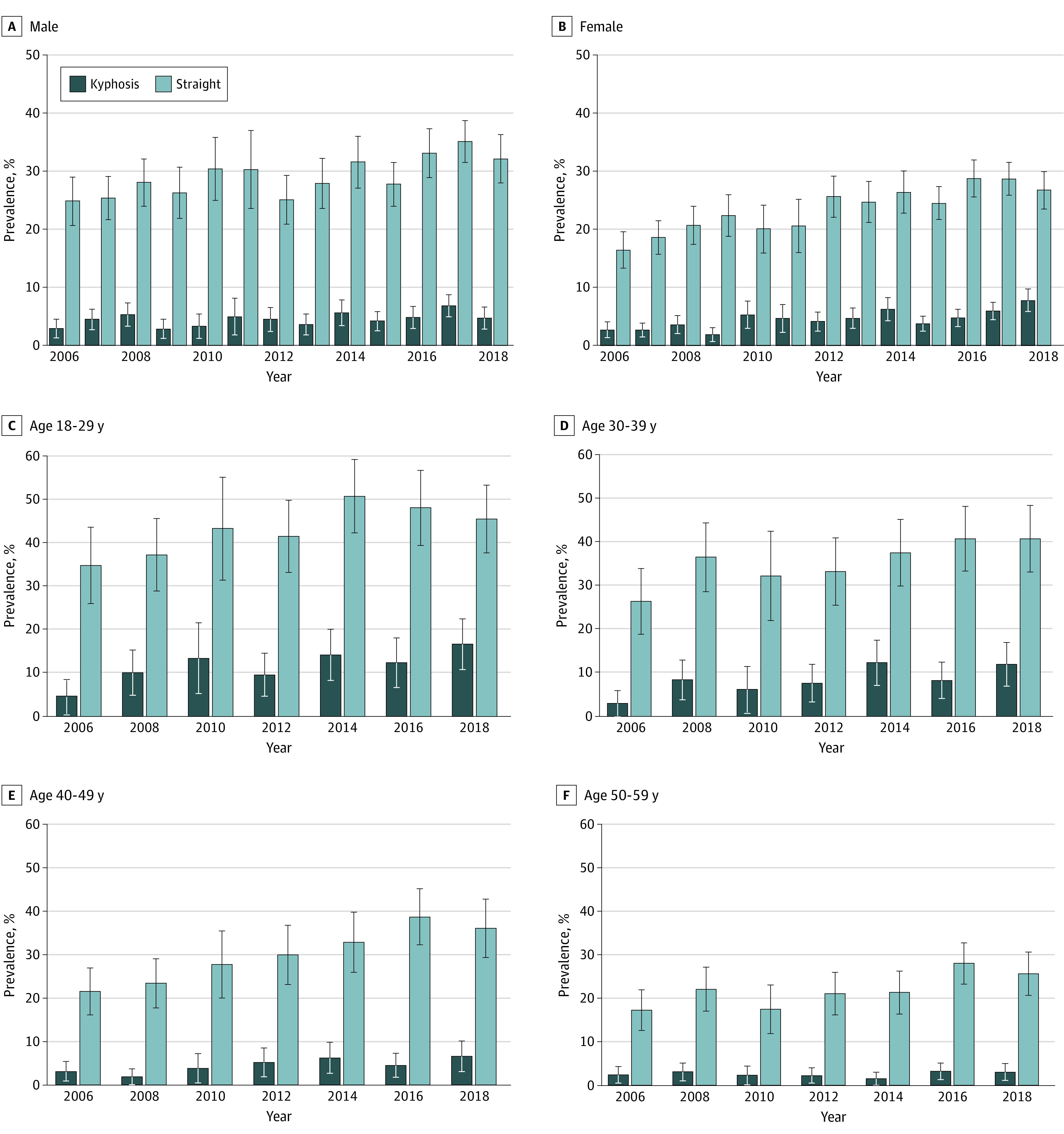
Kyphotic and Straight Cervical Curvature Prevalence According to Sex and Age Between 2006 and 2018 Error bars indicate 95% CIs.

For all periods, individuals 18 to 39 years of age had the highest prevalence of kyphotic and straight curvature (Table 2). From 2006 to 2018, the largest increase in straight curvature prevalence was observed in the 40- to 49-year age group (from 21.5% [95% CI, 16.1%-26.9%] to 36.0% [95% CI, 29.3%-42.7%]) followed by the 30- to 39-year age group (14.3% increase) and 18- to 29-year age group (10.7% increase). The largest increase in kyphotic curvature prevalence was observed in the 18- to 29-year age group (from 4.5% [95% CI, 0.6%-8.3%] to 16.7% [95% CI, 10.8%-22.5%]) followed by the 30- to 39-year (8.9% increase) and 40- to 49-year (3.5% increase) age groups (Figure 2).

**Figure 3.**
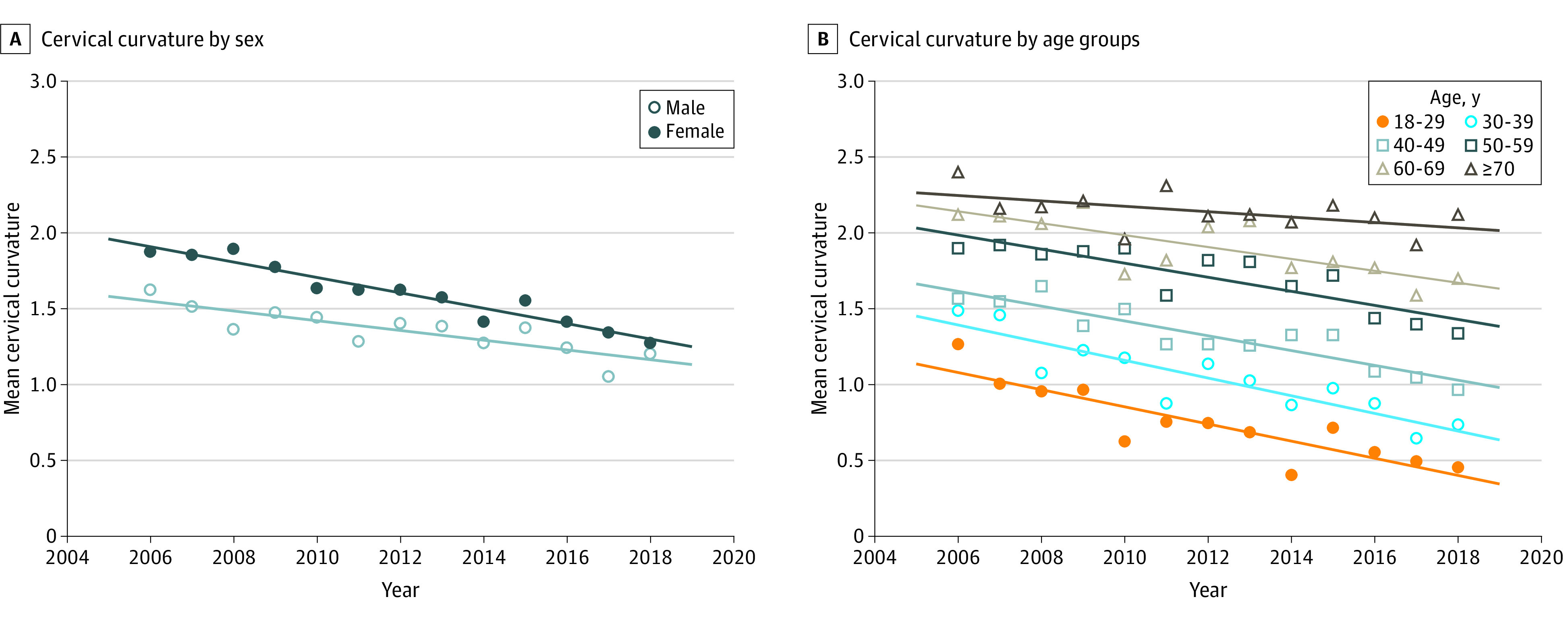
Trends in Mean Cervical Curvature According to Sex and Age Groups in South Korean Adults Between 2006 and 2018 Horizontal line indicates linear regression.

In a linear regression model of the mean cervical curvature from 2006 to 2018 (eTable 3 in the Supplement), a downward trend was observed in men and women, with the decrease more pronounced for women (β = −0.05; 95% CI, −0.06 to −0.04) compared with men (β = −0.03; 95% CI, −0.05 to −0.02) (*P* < .001 for both). For age-specific analysis, the decrease in the annual mean cervical curvature was the highest in younger adults belonging to the age groups of 18 to 29 years and 30 to 39 years (β = −0.06; 95% CI, −0.08 to −0.04; *P* < .001). All age groups younger than 70 years had a significant decrease (eTable 3 in the Supplement), with the magnitude of decrease becoming steeper in successively younger generations (18-29 years of age: −0.06; 95% CI, −0.08 to −0.04; 30-39 years of age: −0.06; 95% CI, −0.08 to −0.04; 40-49 years of age: −0.05; 95% CI, −0.06 to −0.03; 50-59 years of age: −0.05; 95% CI, −0.06 to −0.03 [all *P* < .001]; and 60-69 years of age: −0.04; 95% CI, −0.06 to −0.02; *P* = .002) (Figure 3). Accordingly, the linear mixed-effects model analysis of individuals with repeated measures over time demonstrated consistent results (eTable 4 in Supplement), with a significant decrease across both sex (women: −0.06; 95% CI, −0.08 to −0.05; men: −0.04; 95% CI, −0.06 to −0.02) and all age groups (18-29 years of age: −0.10; 95% CI, −0.16 to −0.04; 30-39 years of age: −0.12; 95% CI, −0.16 to −0.08; 40-49 years of age: −0.07; 95% CI, −0.10 to −0.04; 50-59 years of age, −0.06; 95% CI, −0.08 to −0.04; 60-69 years of age, −0.06; 95% CI, −0.08 to −0.03; and ≥70 years of age, −0.05; 95% CI, −0.08 to −0.02).

## Discussion

In this cross-sectional study, among a cohort of patients who underwent cervical spine radiography, the degree of lordotic curve decreased considerably from 2006 to 2018, with a steeper decrease in progressively younger adults and women. The decrease was highly anticipated and strongly associated with the increase in patients with forward neck-head posture from 1 916 556 (in 2015) to 2 241 679 (in 2019) in South Korea.^29^ The strong association between abnormal head postures among computer and mobile device users that induces sustained flexed neck positions has been acknowledged in previous studies.^30,31,32,33^ By the end of 2019, there were more than 4.38 billion active internet users worldwide. In 2018, South Korea was highly ranked for smartphone penetration (68%) after the UK (82%), the Netherlands (79%), Sweden (79%), Germany (79%), and the US (77%).^34^ A systematic review^10^ reported that the head flexion angle was larger when individuals were text messaging compared with when they were browsing the web and watching videos and that NSP is related to the duration of the mobile device use.^5^ In addition, a previous study^35^ that found that neck-related musculoskeletal conditions were more prevalent for handheld device users by 17.3% to 67% than any other region of the spine suggests practical implications for the mobile device–centric society.

This study suggests that the decline in mean cervical curvature from 2006 to 2018 was definite for men and women and more definite in women. The prevalence of lordotic curvature was generally higher in women compared with men, whereas existing literature suggests lower cervical lordosis in women.^36^ However, the decrease was more pronounced in women, and the difference in the mean cervical coefficients between the 2 groups provided evidence of narrowing in the later years of the study. This rapid decrease for women might be associated with the increase in mobile-centric environments in the past decade and higher levels of smartphone addiction and use time for women.^37,38^ Increasing evidence supports NSP more frequently being reported among women,^5,39^ as well as pain related to the use of electronic devices.^40^

Regarding the age groups, previous cross-sectional studies^36,41^ suggested a higher rate of loss in cervical lordosis in younger adults compared with those studies^12,13^ that reported the lack of any significant association between age and lordotic cervical curvature. This study confirms that younger generations had the lowest mean cervical curvature during the study. From 2006 to 2018, the mean of the cervical curvature and trends of individuals’ cervical curves for age groups of 18 to 29 and 30 to 39 years have shown the steepest decreases among all age groups. In 2017, internet use rates by age in South Korea were more than 99.7% for those 10 to 40 years of age, 98.7% for those in their 50s, 82.5% for those in their 60s, and 31.8% for those 70 years and older.^42^ This finding could be associated with the progressive increase in the prevalence of abnormal cervical curves in younger age groups. The concerning prevalence of abnormal curvature in the 18- to 29-year age group in later years (60.0%-62.2% in 2017-2018) indicates that the loss of cervical lordosis is becoming more frequent, thereby providing much needed evidence to support the theory of “text neck” (stress injury and pain in the neck resulting from excessive use of handheld devices) as a worsening epidemic of the modern era.^39,43^ Considering the increasing neck pain in younger people, the burden of abnormal cervical curvature might increase in the future, potentially reducing or reversing the progress achieved in decreasing chronic morbidity during the next several decades.

This analysis suggests that the critical age for straight and kyphotic cervical curves was in the age groups younger than 40 years. Furthermore, the steep increases in the rates of abnormal curvature were observed for not only young adults but also middle-aged adults 40 to 69 years of age, especially with straight cervical curvature. Even if public health care policy makers are aware of these risks, in-depth evidence that fully analyzes population-based, long-term observations was not available. Public health information on work-related upper-limb disorders is prevalent^44,45^; however, community-level regulatory interventions or social environments for facilitating activities to avoid dysfunctional movements of the cervical spine are largely absent. The active promotion of interventions, particularly government recommendations on mobile phone use^46^ and modifiable risk factors not limited to children but also for young adults, is necessary to encourage awareness.

### Strengths and Limitations

This study has strengths, including the use of a fully automated DLS pipeline to investigate a large-scale population in South Korea. To the best of our knowledge, this is the largest extensive study in the published literature, spanning 13 years. The proposed DLS offers future advantages to assist routine clinical evaluation,^47^ surgical interventions, and pain management.^48^ In addition, consistency of measurement, near-instantaneous assessment, and cost-effectiveness of DLS will facilitate larger-scale, population-based epidemiologic studies and clinical trials.

This study has limitations. First, inferences related to the cause and effect of environmental and social factors to the cervical curvature cannot be made. Additional studies on physical, psychosocial, and personal risk factors, including surveys on time spent using the internet or devices, are required to assess the direct relationship with the cervical curvature. Second, this population-based study evaluated incidences from a South Korean cohort within a single tertiary center. Third, the data only provide information on patients who visited the hospital and underwent standing cervical radiography because of neck pain, upper arm pain, osteoporosis, and degenerative changes. Thus, this trend analysis is based on a clinical cohort and does not reflect community settings.

## Conclusions

The findings suggest that automated DLS can be developed to accurately measure the convexity of the cervical spine and conduct a large, population-based epidemiologic study. From 2006 to 2018, a significant loss of normal cervical lordotic curvature was reported, with a steeper decrease in progressively younger adults and women. Associated ergonomics concerning NSP should be addressed further to minimize the medical and economic burden on the health care systems while obviating the need for the development of practical interventions aimed at neck posture correction.

## Notes

Supplement. **eMethods 1.** The Inclusion and Exclusion Criteria for the Study **eMethods 2.** Exclusion Criteria for the Selection of Cases for Trend Analysis **eMethods 3.** Technical Details of DLS Development: Label Preparation, Image Preprocessing, and Training Process **eTable 1.** Summary Statistics of Patients in the Training, Validation, and Test Set Used for Training of the DLS (Deep Learning System) **eTable 2.** Consistency Between the Manual and Automated Cervical Curvature Measurement **eTable 3.** Trends in Mean Cervical Curvature by Age and Sex (2006 to 2018) **eTable 4.** Trend of Cervical Curvature Associated with Gender and Age from Linear-Mixed Effects Model (2006-2018) **eFigure 1.** Deep Learning System (DLS): The Convolutional Neural Network for Cervical Curvature Segmentation Using a U-Net Architecture **eFigure 2.** Example of Cartesian Axis References Used for Measurement of the Cervical Curve **eFigure 3.** Representative Results of Anterior Vertical Curvature Segmentation
