# Supplementary material for: Temporal Trends in Cervical Spine Curvature of South Korean Adults Assessed by Deep Learning System Segmentation, 2006-2018
Source: JAMA Netw Open. 2020 Oct 15;3(10):e2020961. doi: 10.1001/jamanetworkopen.2020.20961 (PMC7563081; doi:10.1001/jamanetworkopen.2020.20961)
Supplement: Supplement. — eMethods 1. The Inclusion and Exclusion Criteria for the Study eMethods 2. Exclusion Criteria for the Selection of Cases for Trend Analysis eMethods 3. Technical Details of DLS Development: Label Preparation, Image Preprocessing, and Training Process eTable 1. Summary Statistics of Patients in the Training, Validation, and Test Set Used for Training of the DLS (Deep Learning System) eTable 2. Consistency Between the Manual and Automated Cervical Curvature Measurement eTable 3. Trends in Mean Cervical Curvature by Age and Sex (2006 to 2018) eTable 4. Trend of Cervical Curvature Associated with Gender and Age from Linear-Mixed Effects Model (2006-2018) eFigure 1. Deep Learning System (DLS): The Convolutional Neural Network for Cervical Curvature Segmentation Using a U-Net Architecture eFigure 2. Example of Cartesian Axis References Used for Measurement of the Cervical Curve eFigure 3. Representative Results of Anterior Vertical Curvature Segmentation [file jamanetwopen-e2020961-s001.pdf]

Supplementary Online Content

Shin Y, Han K, Lee YH. Temporal trends in cervical spine curvature of South Korean adults assessed by deep learning system segmentation, 2006-2018. *JAMA Netw Open*. 2020;3(10):e2020961. doi:10.1001/jamanetworkopen.2020.20961

eMethods 1. The Inclusion and Exclusion Criteria for the Study

eMethods 2. Exclusion Criteria for the Selection of Cases for Trend Analysis

eMethods 3. Technical Details of DLS Development: Label Preparation, Image Preprocessing, and Training Process

eTable 1. Summary Statistics of Patients in the Training, Validation, and Test Set Used for Training of the DLS (Deep Learning System)

eTable 2. Consistency Between the Manual and Automated Cervical Curvature Measurement

eTable 3. Trends in Mean Cervical Curvature by Age and Sex (2006 to 2018)

eTable 4. Trend of Cervical Curvature Associated with Gender and Age from Linear-Mixed Effects Model (2006-2018)

eFigure 1. Deep Learning System (DLS): The Convolutional Neural Network for Cervical Curvature Segmentation Using a U-Net Architecture

eFigure 2. Example of Cartesian Axis References Used for Measurement of the Cervical Curve

eFigure 3. Representative Results of Anterior Vertical Curvature Segmentation

This supplementary material has been provided by the authors to give readers additional information about their work.

© 2020 Shin Y et al. *JAMA Network Open.*

eMethods 1. The Inclusion and Exclusion Criteria for the Study.

The inclusion criteria were: (1) cervical lateral radiographs; (2) standing position radiographs associated with neck pain, upper arm pain, osteoporosis, and degenerative changes; (3) from inpatient and outpatient settings. The exclusion filtering criteria were as follows: the (1) “postoperative status,” “interbody fixation,” “interbody fusion,” “anterior cervical discectomy and fusion (ACDF),” “spinal fusion,” and “laminoplasty” in radiological reports; (2) flexion or extension view of cervical dynamogram; (3) pediatric patients under the age of 18; and (4) radiographs from specialized clinics including cerebral palsy and department of clinical genetics; (5) radiographs repeatedly measured in the same individual on the same year. Additional radiographic review was performed by a musculoskeletal radiologist with 12 years of experience to exclude a total of 2358 patients with devices in radiographs; patients with surgical devices (n=1943), patients with clips (n=414), patients with clips (n=1).

© 2020 Shin Y et al. *JAMA Network Open.*

eMethods 2. Exclusion Criteria for the Selection of Cases for Trend Analysis

An age and gender-blinded review were proceeded by a board-certified musculoskeletal radiologist (> 12 years of clinical radiology experience) and reviewer with two years of musculoskeletal imaging experience. The reviewers reviewed the DLS-segmentation superimposed in the original lateral radiograph and excluded patients with false segmentation in their first-visit radiographs or follow-up radiographs by consensus. The exclusion process for false segmentation are as follows: For first-visit patients; (a) Cervical curvature segmentation disconnection (n=27), (b) segmentation curvature not fully covering anterior vertebrae (n=16), (c) false segmentation outside of the cervical spinal region (n=5). For patients with follow-up radiographs; (a) Cervical curvature segmentation disconnection (n=1), (b) segmentation curvature not fully covering anterior vertebrae (n=1)

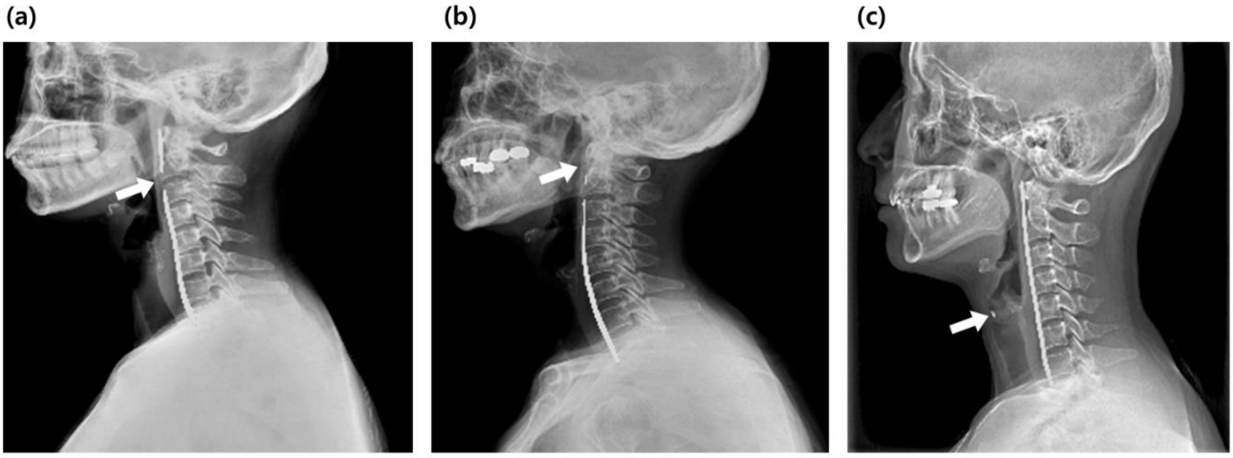

© 2020 Shin Y et al. *JAMA Network Open.*

eMethods 3. Technical Details of DLS Development: Label Preparation, Image Preprocessing, and Training Algorithm.

To generate the ground truth segmentation label, the anterior vertical lines starting at the anterior point of the odontoid process and ending at the anterior point of the body of C7 were manually annotated by an expert musculoskeletal radiologist (> 12 years of clinical radiology experience) using MATLAB (MathWorks, Natick, MA).

Before training, the model the data is preprocessed in the following steps: (1) any black band and the border is cropped from the edges of the image, (2) images are resized into 256×256 pixels, (3) the mean over all pixels in the training dataset is subtracted, and pixel values are divided by their standard deviation. The mean and the standard deviation are saved, and the same normalization is applied in the prediction stage. During the training, data augmentation is performed with rotation ($-20°$ to $20°$), translation shift ($0 - 5\%$ of image size in horizontal and vertical axes), and zoom ($0° - 5°$). The pixel-wise weighted loss and Dice loss was optimized with Adam solver at the mini-batch size of 16 and a learning rate of 0.0001. We selected the number of training epochs for 400 epochs maximum using early stopping on a validation set.

The sample size of 325 cervical radiographs was selected by empirically increasing the number of images included in the training set. The pair of cervical lateral radiographs and the corresponding ground truth segmentation images was increased progressively until a satisfactory segmentation output of radiologist review and convergence in the accuracy was achieved.

© 2020 Shin Y et al. *JAMA Network Open.*

eTable 1. Summary Statistics of Patients in the Training, Validation, and Test Set Used for Training of the DLS (Deep Learning System).

**Categories**	**Training**	**Validation**	**Test**
Patients, No	325	130	130
Sex			
Female	198 (60.9%)	76 (58.5%)	67 (51.5%)
Male	127 (39.1%)	54 (41.5%)	63 (48.5%)
Mean (SD)	52.5 (15.9)	57.2 (17.4)	57.8 (16.7)
Range	18 to 90	18 to 89	18 to 91

© 2020 Shin Y et al. *JAMA Network Open.*

eTable 2. Consistency Between the Manual and Automated Cervical Curvature Measurement. CI: confidence interval, ICC: intraclass correlation coefficient, RSME: root-mean-squared error

Year	ICC (95% CI)	RMSE
2006	0.974 (0.947, 0.988)	0.300
2007	0.987 (0.973, 0.994)	0.250
2008	0.985 (0.968, 0.993)	0.260
2009	0.990 (0.980, 0.995)	0.221
2010	0.980 (0.958, 0.990)	0.268
2011	0.989 (0.977, 0.995)	0.197
2012	0.988 (0.975, 0.994)	0.222
2013	0.991 (0.982, 0.996)	0.208
2014	0.988 (0.976, 0.994)	0.200
2015	0.988 (0.975, 0.994)	0.206
2016	0.986 (0.971, 0.993)	0.199
2017	0.992 (0.984, 0.996)	0.192
2018	0.993 (0.986, 0.997)	0.205
Total	0.987 (0.974, 0.994)	0.225

© 2020 Shin Y et al. *JAMA Network Open.*

eTable 3. Trends in Mean Cervical Curvature by Age and Sex (2006 to 2018)

	Mean Cervical Curvature (SD)								
	Sex			Age Range, y					
Year	Mean	Male	Female	18-29	30-39	40-49	50-59	60-69	≥70
2006	1.76 (1.37)	1.62 (1.31)	1.87 (1.40)	1.26 (1.25)	1.48 (1.30)	1.56 (1.28)	1.89 (1.38)	2.11 (1.34)	2.39 (1.41)
2007	1.70 (1.41)	1.51 (1.36)	1.85 (1.43)	1.00 (1.34)	1.45 (1.33)	1.54 (1.31)	1.91 (1.29)	2.10 (1.52)	2.15 (1.39)
2008	1.65 (1.51)	1.36 (1.35)	1.89 (1.59)	0.95 (1.40)	1.07 (1.49)	1.64 (1.34)	1.85 (1.44)	2.05 (1.46)	2.16 (1.75)
2009	1.64 (1.36)	1.47 (1.34)	1.77 (1.37)	0.96 (1.34)	1.22 (1.19)	1.38 (1.42)	1.87 (1.20)	2.19 (1.35)	2.20 (1.37)
2010	1.55 (1.49)	1.44 (1.29)	1.63 (1.63)	0.62 (1.38)	1.17 (1.45)	1.49 (1.38)	1.89 (1.41)	1.72 (1.61)	1.95 (1.34)
2011	1.49 (1.43)	1.28 (1.36)	1.62 (1.47)	0.75 (1.28)	0.87 (1.54)	1.26 (1.09)	1.58 (1.35)	1.81 (1.52)	2.30 (1.45)
2012	1.53 (1.41)	1.40 (1.30)	1.62 (1.48)	0.74 (1.39)	1.13 (1.38)	1.26 (1.26)	1.81 (1.39)	2.03 (1.19)	2.10 (1.42)
2013	1.49 (1.42)	1.38 (1.32)	1.57 (1.49)	0.68 (1.60)	1.02 (1.34)	1.25 (1.25)	1.80 (1.36)	2.07 (1.25)	2.11 (1.25)
2014	1.35 (1.38)	1.27 (1.26)	1.41 (1.46)	0.40 (1.35)	0.86 (1.35)	1.32 (1.30)	1.64 (1.20)	1.76 (1.23)	2.06 (1.37)
2015	1.48 (1.32)	1.37 (1.23)	1.55 (1.38)	0.71 (1.36)	0.97 (1.23)	1.32 (1.14)	1.71 (1.25)	1.80 (1.25)	2.17 (1.37)
2016	1.34 (1.39)	1.24 (1.27)	1.41 (1.46)	0.55 (1.21)	0.87 (1.32)	1.08 (1.20)	1.43 (1.33)	1.76 (1.30)	2.09 (1.52)
2017	1.22 (1.42)	1.05 (1.27)	1.34 (1.50)	0.49 (1.37)	0.64 (1.26)	1.04 (1.45)	1.39 (1.24)	1.58 (1.40)	1.91 (1.42)
2018	1.24 (1.37)	1.20 (1.26)	1.27 (1.44)	0.45 (1.33)	0.73 (1.36)	0.96 (1.19)	1.33 (1.20)	1.69 (1.21)	2.11 (1.45)
β (95% CI)	-0.04 (-0.05,-0.03)	-0.03 (-0.05,-0.02)	-0.05 (-0.06,-0.04)	-0.06 (-0.08,-0.04)	-0.06 (-0.08,-0.04)	-0.05 (-0.06,-0.03)	-0.05 (-0.06,-0.03)	-0.04 (-0.06,-0.02)	-0.02 (-0.04,-0.00)
P for trend	<.001	<.001	<.001	<.001	<.001	<.001	<.001	.002	.055

© 2020 Shin Y et al. *JAMA Network Open.*

eTable 4. Trend of Cervical Curvature Associated with Gender and Age from Linear-Mixed Effects Model (2006-2018).

	Sex		Age					
	Male	Female	18-29	30-39	40-49	50-59	60-69	≥70
β (95% CI)	-0.04 (-0.06,-0.02)	-0.06 (-0.08,-0.05)	-0.1 (-0.16, -0.04)	-0.12 (-0.16, -0.08)	-0.07 (-0.1, -0.04)	-0.06 (-0.08, -0.04)	-0.06 (-0.08, -0.03)	-0.05 (-0.08, -0.02)
P for trend	<.001	<.001	<.001	<.001	<.001	<.001	.004	.003

© 2020 Shin Y et al. *JAMA Network Open.*

eFigure 1. Deep Learning System (DLS): The Convolutional Neural Network for Cervical Curvature Segmentation Using a U-Net Architecture.

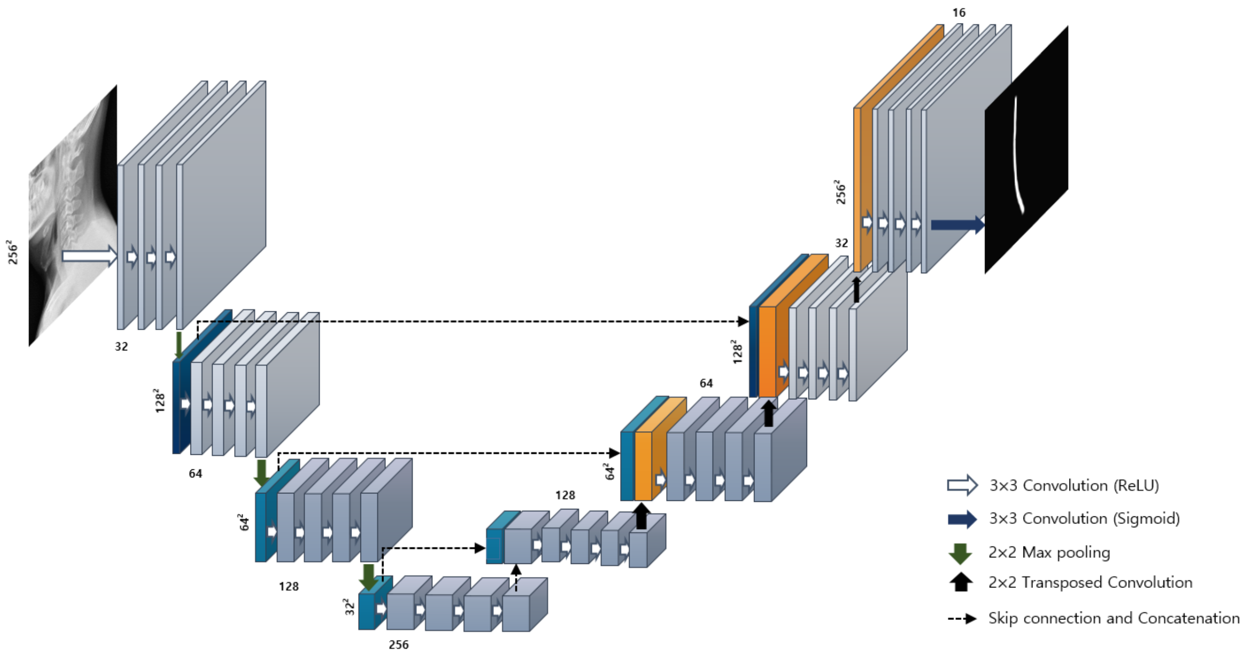

The deep learning process is divided into two parts: an encoder that extracted the features while reducing the resolution of the feature maps, and a decoder that expands the encoding to a full-resolution segmentation image. The model adapts 33 convolutional (C) layers with 3 × 3 convolutional kernel, where 2 × 2 max-pooling (MP) layers us applied for down-sampling in the encoder. The decoder adapts a sequence of 3 × 3 transposed convolutions for up-sampling, and the up-sampled features are fused with the corresponding feature maps from the encoder with the same resolution. The up-sampled features are then followed by a 1 × 1 convolution that outputs the pixel-wise classification creating a mask for anterior vertical line.

© 2020 Shin Y et al. *JAMA Network Open.*

eFigure 2. Example of Cartesian Axis References Used for Measurement of the Cervical Curve.

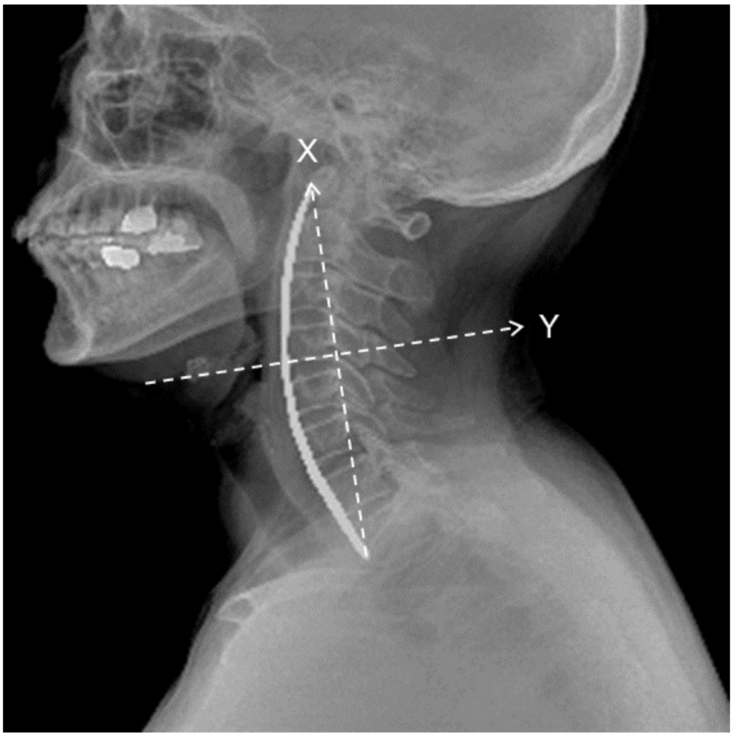

© 2020 Shin Y et al. *JAMA Network Open.*

eFigure 3. Representative Results of Anterior Vertical Curvature Segmentation. Curvature Coefficients Obtained from Second-Order Polynomial Regression are Indicated the Corresponding Lateral Radiographs. The Lateral Radiographs are Classified into Lordosis (Curvature Coefficient > 0.8), Straight (-0.8 < Curvature Coefficient < 0.8), and Kyphosis (Curvature Coefficient < - 0.8).

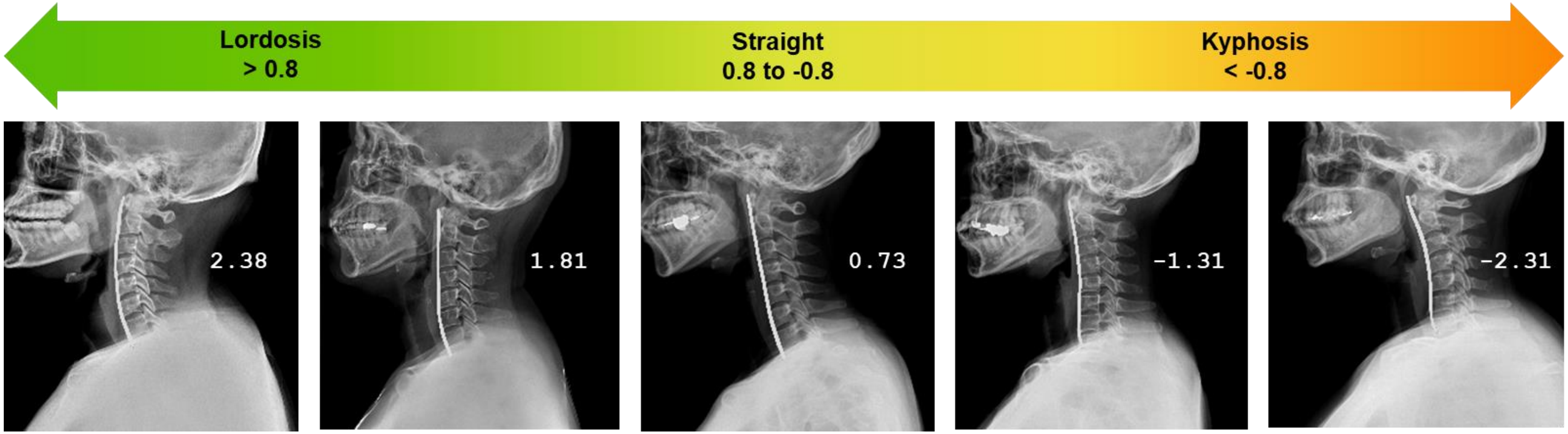

© 2020 Shin Y et al. *JAMA Network Open.*
